# Nonlinear association between body roundness index and metabolic dysfunction associated steatotic liver disease in nondiabetic Japanese adults

**DOI:** 10.1038/s41598-025-99540-5

**Published:** 2025-05-02

**Authors:** Cheng Huang, Zhichao Gao, Zhenxia Huang, Junfeng Xu

**Affiliations:** 1 Department of Colorectal Surgery, First People’s Hospital of Xiaoshan District, Hangzhou, 311200 Zhejiang China; 2 Department of Neurosurgery, First People’s Hospital of Xiaoshan District, Hangzhou, 311200 Zhejiang China; 3 Department of Infectious Diseases, First People’s Hospital of Xiaoshan District, Hangzhou, 311200 Zhejiang China

**Keywords:** Body roundness index (BRI), Metabolic dysfunction associated steatotic liver disease (MASLD), Obesity, Non-diabetic Japanese, Nonlinear, Diseases, Endocrinology, Medical research

## Abstract

The global rise in obesity and diabetes has been paralleled by a rising incidence of metabolic dysfunction-associated steatotic liver disease (MASLD). Although previous studies have explored the association between body roundness index (BRI) and MASLD, the specific relationship in non-diabetic Japanese adults requires further investigation. This study analyzed data from 15,299 participants enrolled in the NAGALA cohort (2004–2015) to explore the association between BRI and MASLD through multivariable logistic regression, stratified analysis, and restricted cubic spline modeling. The prevalence of MASLD was 14.46%, with 13.73% occurring in non-obese individuals (BMI < 30). After adjusting for all confounding factors, BRI demonstrated a significant association with MASLD, yielding an adjusted odds ratio of 1.72 (95% CI 1.48–1.99). The restricted cubic spline model revealed a nonlinear relationship, with an inflection point at 3.06. Stratified analyses revealed stronger associations in individuals with lower BMI (≤ 24 kg/m^2^).

**Supplementary Information:**

The online version contains supplementary material available at 10.1038/s41598-025-99540-5.

## Introduction

Metabolic dysfunction-associated steatotic liver disease (MASLD) highlights the crucial role of metabolic dysfunction in the development and progression of the disease^1^. Beyond indicating hepatic lesions, MASLD serves as a critical marker of systemic metabolic disturbances. Emerging evidence indicates that around 99% of non-alcoholic fatty liver disease (NAFLD) individuals also meet MASLD criteria, showing high concordance between the two definitions^2,3^. Recently, there has been a dramatic increase in the incidence of MASLD, which has coincided with worldwide prevalence of obesity and diabetes^4^. MASLD is commonly recognized as the primary contributor to chronic liver disease globally, affecting approximately 38% of adults and between 7 and 14% of children^5,6^.

The pathophysiology of MASLD encompasses various factors, including insulin signaling disruptions, irregular fatty acid metabolism, and the activation of inflammatory processes^7^. These factors collectively promote insulin resistance, obesity, and exacerbate metabolic dysfunction^8^. MASLD can worsen steatosis into non-alcoholic steatohepatitis, cirrhosis, and hepatocellular cancer^9^. A further point to consider is that MASLD is closely linked to metabolic syndrome and diabetes^10^. Thus, precise detection and effective intervention for populations at high risk of MASLD are key priorities in current research. Obesity, particularly visceral obesity, is strongly correlated with MASLD and is considered a key predictive factor for the disease^11,12^. Among Asians, the prevalence was 23.5% for non-obese MASLD and 40.7% for obese MASLD^13^. Although BMI is extensively used to measure obesity, it does not completely capture the distribution of fat. To address this limitation, the body roundness index (BRI) was introduced by Thomas et al.^14^. Unlike BMI, which relies solely on weight and height, BRI uses waist circumference as a core metric, offering enhanced sensitivity to visceral fat accumulation^15^. BRI has been explored in relation to conditions including diabetes, cardiovascular diseases, metabolic syndrome^15–18^. Due to the significant correlation between obesity and MASLD, BRI has emerged as a potential predictor of MASLD presence in the American population^19^.

However, the association between BRI and MASLD is still insufficiently explored, particularly in non-diabetic populations. This study aims to evaluate the association between BRI and MASLD in non-diabetic Japanese adults.

## Methods

### Data source and study population

This research utilized data from the NAGALA study, a longitudinal analysis focused on NAFLD in the Gifu Area. The original research began in 1994 at Murakami Memorial Hospital in Japan. This project conducts comprehensive medical evaluations, doing more than 8000 assessments each year. Around 60% of individuals undergo one to two examinations per year, enabling a thorough longitudinal analysis of health data.

The dataset for this research includes individuals who underwent several health assessments from 2004 to 2015. A total of 20,944 participants were initially identified, all of whom were free from diabetes at baseline. Data were sourced from the Dryad Digital Repository. The NAGALA project received ethical approval from the Clinical Research Ethics Committee of Murakami Memorial Hospital, and all participants provided informed consent for their data to be used in the research^20^. To ensure the integrity of the analysis, several exclusion criteria were applied. Participants with pre-existing liver illnesses, including viral hepatitis, those with excessive alcohol intake, and persons on medication at baseline were excluded from the dataset. Furthermore, individuals diagnosed with diabetes were excluded. Subsequent to the application of these criteria, the final study cohort comprised 15,464 participants, encompassing 7034 women and 8430 men.

**Fig. 1:**
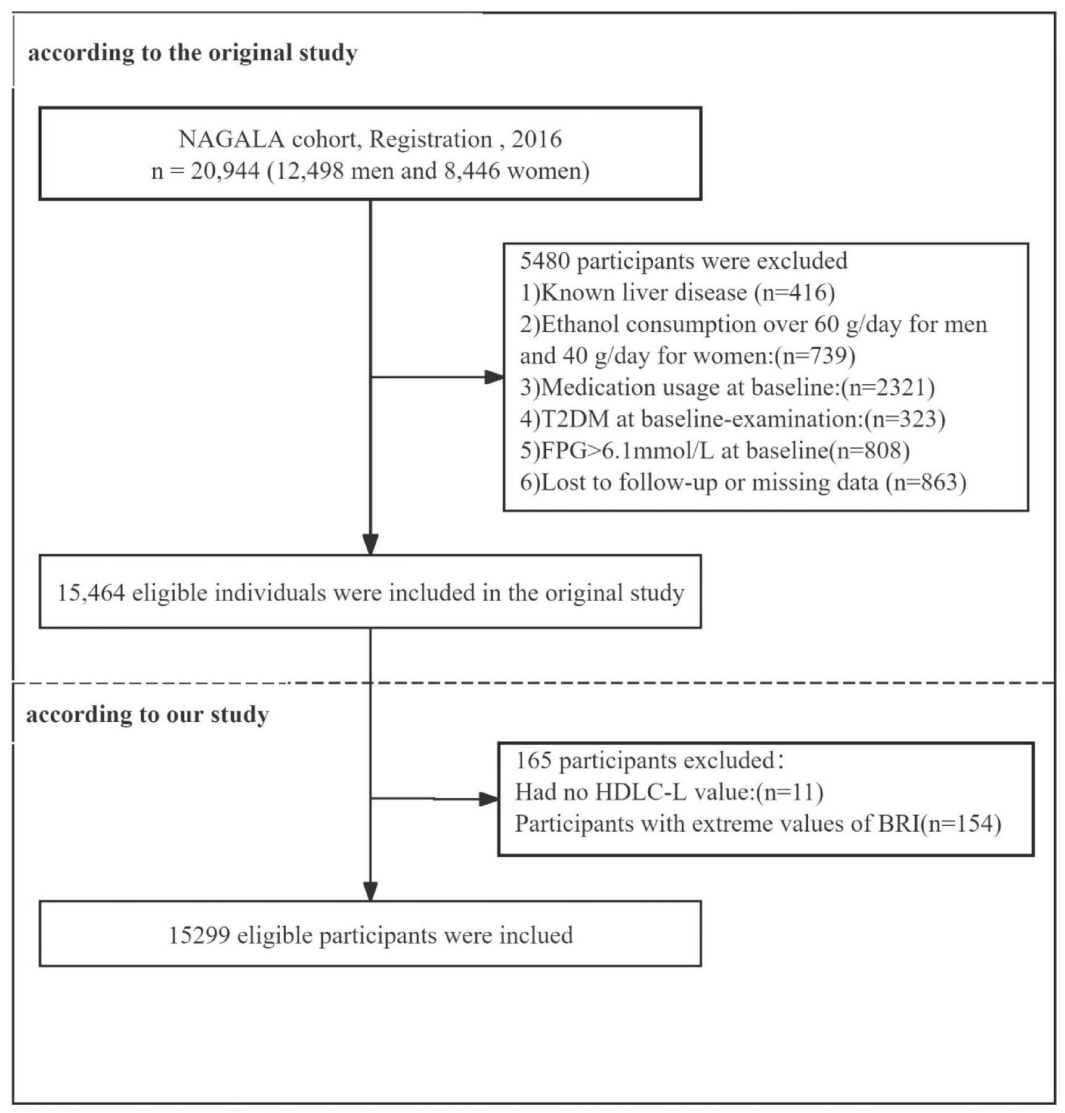
Flowchart of the sample selection.

Participants without high-density lipoprotein cholesterol (HDL-C) data were eliminated from this study (n = 11). Furthermore, participants with extreme BRI values were excluded (n = 154). Extreme BRI values defined as BRI < mean − 3 × SD or BRI > mean + 3 × SD. Finally, 15,299 participants were involved in the study (Fig. 1).

### Data collection

This study retrieved various baseline data from the NAGALA database, encompassing fundamental demographic information and health markers of the individuals^20^. Data collection encompassed age, gender, BMI, waist circumference (WC), blood pressure, HDL-C, liver enzymes, glycated hemoglobin (HbA1c), triglycerides (TG), total cholesterol (TC), and fasting plasma glucose (FPG). After fasting for at least 8 h, each participant had blood drawn for analysis, which was subsequently evaluated using an automatic biochemical analyzer. The lifestyle characteristics of participants were evaluated using standardized questionnaires that included smoking and alcohol consumption, exercise routines, and other relevant aspects. According to smoking habits, participants were classified into three categories: never, former, and current. Drinking status was characterized based on alcohol intake in the preceding month^21^. Exercise habits were characterized by participation in physical exercise a minimum of once per week. Specialists in gastroenterology diagnosed fatty liver according to findings of abdominal ultrasound examinations performed on patients^22^. Metabolic syndrome (MetS) is calculated based on the criteria set by the National Cholesterol Education Program^23^. MASLD is defined in the 2020 International Expert Consensus Statement, which describes its diagnostic criteria as the presence of hepatic steatosis alongside metabolic abnormalities^24^.

The BRI was computed as follows^14^:

$$BRI = 364.2 - 365.5\sqrt {1 - \left( {\frac{WC}{{2\Pi }}} \right)^{2} \div \left( {0.5{\text{height}}} \right)^{2} }$$

### Statistical analysis

Continuous data are typically presented as mean ± standard deviation or median with interquartile range, while categorical data are generally reported as counts and proportions. Chi-square tests were utilized for the analysis of categorical data. In order to compare continuous variables between groups, we utilized analysis of variance (ANOVA) and Kruskal–Wallis tests. We estimated the variance inflation factor (VIF) in our multivariate models in order to manage the possibility of collinearity among the covariates. We excluded any covariates that had a VIF that was larger than 5 due to the fact that they were collinear (Supplementary Table S1). In addition to this, we carried out univariate logistic regression in order to investigate the potential impact that BRI may have on the occurrence of MASLD. Multivariable logistic regression analysis was conducted to evaluate associations with the dependent variable across three models. Model 1 adjusted for sex, age, BMI, exercise habits, smoking status, drinking status and SBP. Model 2 included HbA1c, ALT, AST, GGT, and FPG based on Model 1. Model 3 further adjusted for TG, TC, HDL-C and MetS.

To validate the robustness of our primary findings across different populations, we performed sensitivity analyses. These analyses were designed to assess the stability and consistency of the results by evaluating various scenarios and assumptions, thereby ensuring that our conclusions remain valid even when accounting for potential variations in the data. To minimize potential fat effects, sensitivity Analysis 1 excluded individuals whose BMI > 24 kg/m^2^. Sensitivity Analysis 2 excluded individuals aged > 50 years, while Sensitivity Analysis 3 excluded participants with MetS. To evaluate whether the association between BRI and MASLD remains significant after accounting for multiple comparisons, Bonferroni correction was applied. The dose–response relationship between BRI and MASLD was assessed using a restricted cubic spline (RCS) regression model with knots placed at the 25th, 50th, and 75th percentiles of the BRI distribution^25^. A two-segment logistic regression approach was utilized to accurately identify the inflection point. Additionally, we performed stratified analyses based on demographic characteristics, exercise patterns, presence of fatty liver, and lifestyle behaviors like smoking and alcohol use, examining potential interactions among these factors. Data analyses were conducted utilizing Empower stats (www.empowerstats.com, X&Y Solutions, Inc., Boston, MA), with statistical significance defined as a *P* value of less than 0.05.

## Results

### Baseline characteristics

The study involved a total of 15,299 participants, where males represented 54.60% of the population. This cohort study revealed a prevalence of MASLD at 14.46%. The prevalence of MASLD in non-diabetic Japanese Adults (BMI < 30) was 13.73%. The study population was categorized into four quartile-based groups (Table 1). The analysis indicated that with the rise in BRI, there were notable increases in participants’ age, BMI, WC, transaminases, TC, TG, HbA1c, FPG, and blood pressure, while HDL-C showed a marked decrease. Moreover, there was a considerable rise in the percentage of males in the elevated BRI categories, with the incidence of MASLD escalating markedly from 0.37% in Q1 to 39.69% in Q4. Exercise habits decreased as BRI increased, and there were observable trends in drinking and smoking statuses, although they were less pronounced than other variables. The findings demonstrate a strong link between elevated BRI and progression of MASLD, along with various aspects of metabolic syndrome and harmful lifestyle behaviors.

**Table 1:** Characteristics of the study population.

Characteristic	Q1 (< 2.09)	Q2 (2.09–2.63)	Q3 (2.63–3.23)	Q4 (≥ 3.23)	*P* value
Subjects, n	3825	3824	3825	3825	
Male, n (%)	1411 (36.89%)	2036 (53.24%)	2417 (63.19%)	2489 (65.07%)	< 0.001
Age (years)	40.81 ± 8.38	42.67 ± 8.48	44.56 ± 8.54	46.76 ± 9.04	< 0.001
BMI (kg/m^2^)	19.02 ± 1.53	21.02 ± 1.52	22.68 ± 1.70	25.35 ± 2.45	< 0.001
WC (cm)	66.28 ± 4.49	73.34 ± 4.18	78.90 ± 4.30	86.33 ± 5.74	< 0.001
ALT (IU/L)	15.18 ± 6.80	17.15 ± 8.27	20.90 ± 18.09	26.09 ± 16.71	< 0.001
AST (IU/L)	16.71 ± 5.71	17.35 ± 6.31	18.66 ± 11.62	20.60 ± 8.52	< 0.001
GGT (IU/L)	14.41 ± 11.20	18.11 ± 15.27	21.98 ± 20.19	26.33 ± 21.24	< 0.001
HDL-C (mmol/L)	1.66 ± 0.39	1.53 ± 0.39	1.39 ± 0.38	1.28 ± 0.34	< 0.001
TC (mmol/L)	4.87 ± 0.81	5.02 ± 0.83	5.20 ± 0.85	5.40 ± 0.87	< 0.001
TG (mmol/L)	0.60 ± 0.33	0.78 ± 0.51	1.01 ± 0.69	1.24 ± 0.80	< 0.001
HbA1c (%)	5.10 ± 0.30	5.14 ± 0.31	5.18 ± 0.32	5.26 ± 0.33	< 0.001
FPG (mmol/L)	4.99 ± 0.39	5.11 ± 0.39	5.22 ± 0.40	5.31 ± 0.39	< 0.001
SBP (mmHg)	106.88 ± 12.80	111.92 ± 13.06	116.31 ± 13.92	122.08 ± 15.13	< 0.001
DBP (mmHg)	66.40 ± 8.82	69.63 ± 9.36	72.95 ± 10.05	76.88 ± 10.47	< 0.001
MetS, n (%)	6 (0.16%)	40 (1.05%)	137 (3.58%)	388 (10.14%)	< 0.001
MASLD, n (%)	14 (0.37%)	127 (3.32%)	554 (14.48%)	1518 (39.69%)	< 0.001
Habit of exercise, n (%)	689 (18.01%)	756 (19.77%)	676 (17.67%)	572 (14.95%)	< 0.001
Drinking status, n (%)					< 0.001
Non	3217 (84.10%)	2872 (75.10%)	2800 (73.20%)	2782 (72.73%)	
Light	335 (8.76%)	498 (13.02%)	458 (11.97%)	449 (11.74%)	
Moderate	218 (5.70%)	331 (8.66%)	386 (10.09%)	415 (10.85%)	
Heavy	55 (1.44%)	123 (3.22%)	181 (4.73%)	179 (4.68%)	
Smoking status, n (%)					< 0.001
Never	2673 (69.88%)	2282 (59.68%)	2008 (52.50%)	1961 (51.27%)	
Past	451 (11.79%)	697 (18.23%)	877 (22.93%)	910 (23.79%)	
Current	701 (18.33%)	845 (22.10%)	940 (24.58%)	954 (24.94%)	

*ALT* alanine aminotransferase, *AST* aspartate aminotransferase, *BMI* body mass index, *TC* total cholesterol, *TG* triglycerides, *HDL-C* high-density lipoprotein cholesterol, *HbA1c* glycated haemoglobin, *FPG* fasting plasma glucose, *SBP* systolic blood pressure, *DBP* diastolic blood pressure, *MetS* metabolic syndrome, *MASLD* metabolic dysfunction associated steatotic liver disease.

### Association between BRI and MASLD

**Fig. 2:**
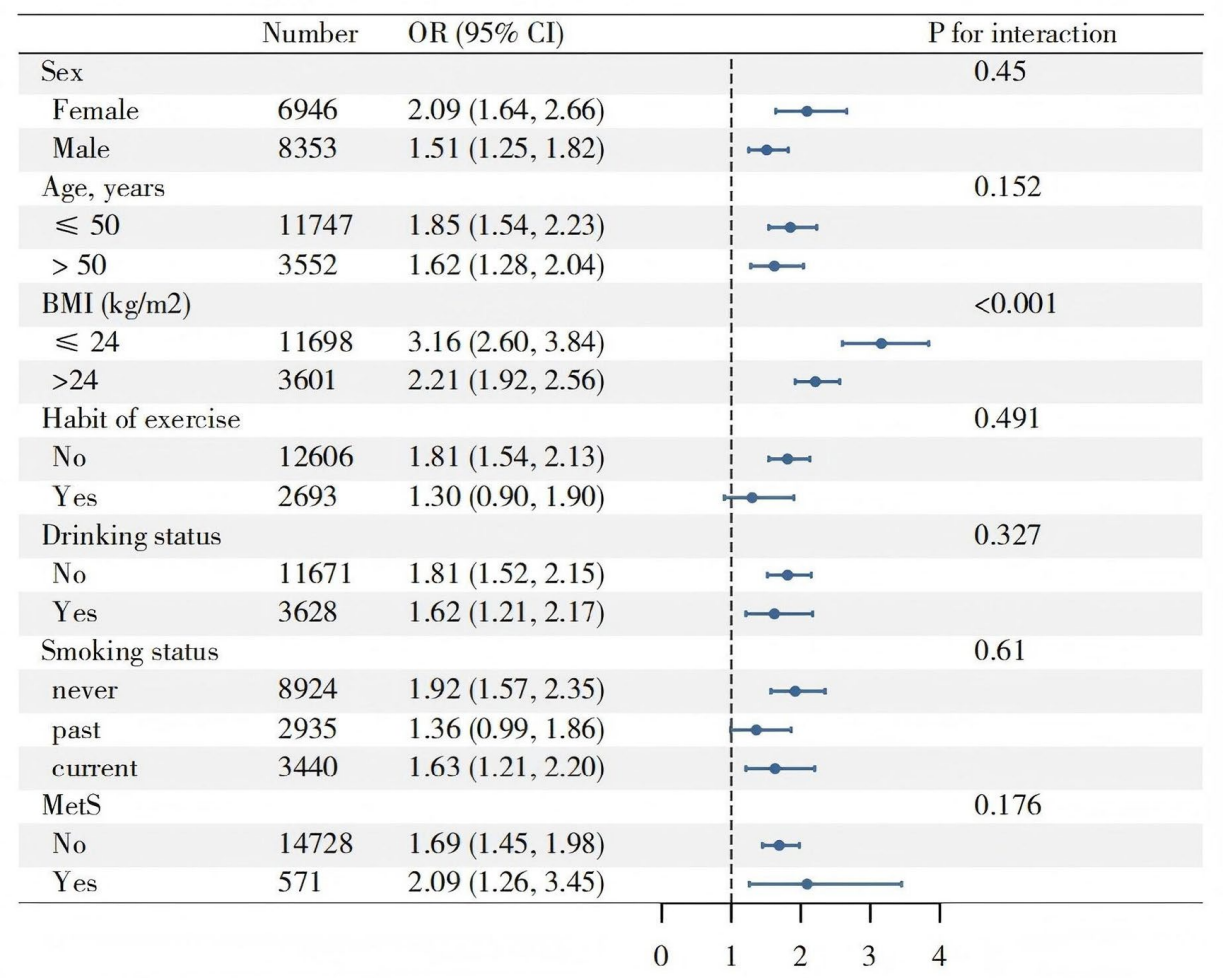
Stratified analysis of associations between BRI and MASLD. Models adjusted for covariates as in Model 3.

The univariate regression analysis, emphasizing notable correlations between all baseline variables and MASLD (Supplementary Table S2). Multivariable analysis demonstrated that the association between BRI and MASLD remained robust even after controlling for confounding variables (Table 2). The model 1 revealed an OR of 2.16 (95% CI 1.89–2.47) for BRI. Following adjustment for covariates, the OR decreased to 1.88 (95% CI 1.63–2.17; Model 2) and then to 1.72 (95% CI 1.48–1.99; Model 3), affirming a strong and positive association between BRI and MASLD. Stratified analysis indicated that BMI significantly influences the association between BRI and MASLD (Fig. 2). The association was more pronounced in participates with a lower BMI (≤ 24 kg/m^2^). In this group, the relationship was notably stronger, with an OR of 3.16 (95% CI 2.60–3.84), compared to the BMI > 24 kg/m^2^ group, which showed an OR of 2.21 (95% CI 1.92–2.56).

**Table 2:** Multivariable regression analyses for the association between BRI and MASLD.

	OR (95% CI)		
	Model 1	Model 2	Model 3
BRI	2.16 (1.89, 2.47)	1.88 (1.63, 2.17)	1.72 (1.48, 1.99)
Q1	Ref	Ref	Ref
Q2	3.57 (2.04, 6.25)	3.10 (1.76, 5.46)	2.51 (1.42, 4.44)
Q3	8.29 (4.79, 14.34)	6.32 (3.63, 10.99)	4.46 (2.55, 7.79)
Q4	12.99 (7.38, 22.86)	9.19 (5.18, 16.30)	6.18 (3.46, 11.03)
*P* trend	< 0.001	< 0.001	< 0.001

Model 1: sex, age, BMI, drinking status, smoking status, habit of exercise and SBP were adjusted.

Model 2: sex, age, BMI, ALT, AST, GGT, habit of exercise, HbA1c, drinking status, smoking status, FPG and SBP were adjusted.

Adjust 3: sex, age, BMI, ALT, AST, habit of exercise, GGT, HDL-C, TC, TG, MetS, HbA1c, drinking status, smoking status, FPG and SBP were adjusted.

### Sensitivity analysis

Three sensitivity analyses were conducted to assess the robustness of the main results and assess the potential influence of specific subgroups (Table 3). Sensitivity analysis 1 excluded participants with a BMI exceeding 24 kg/m^2^ to address the potential confounding effect of adiposity. Individuals aged > 50 years and those with MetS were excluded separately in sensitivity analysis 2 and sensitivity analysis 3. After including the extreme BRI values in the analysis, we performed a multivariable Cox regression analysis to verify the stability and consistency of the results, which demonstrated that the primary findings remained robust (Supplementary Table S3). Finally, after applying Bonferroni correction to account for multiple comparisons, the association between BRI and MASLD remained statistically significant across all subgroup analyses and sensitivity analyses (*P*_adjusted_ < 0.05).

**Table 3:** Sensitivity analysis: the association netwwen BRI and MASLD in different populations.

	OR (95% CI)		
	Sensitivity-1	Sensitivity-2	Sensitivity-3
BRI	3.16 (2.60, 3.84)	1.85 (1.54, 2.23)	1.69 (1.45, 1.98)
Q1	Ref	Ref	Ref
Q2	3.67 (2.07, 6.51)	2.22 (1.22, 4.04)	2.33 (1.32, 4.14)
Q3	8.31 (4.76, 14.51)	3.81 (2.12, 6.87)	4.10 (2.34, 7.18)
Q4	12.78 (7.13, 22.89)	5.65 (3.05, 10.49)	5.75 (3.21, 10.32)

(1) Sensitivity-1: Participants with BMI > 24 kg/m^2^ at baseline were excluded (n = 11,698).

(2) sensitivity-2: Participants diagnosed with age > 50 years at baseline were excluded (n = 11,747).

(3) sensitivity-3: Participants diagnosed with MetS at baseline were excluded (n = 14,728).

Sensitivity-1 was adjusted for sex, age, ALT, AST, habit of exercise, GGT, HDL-C, TC, TG, MetS, HbA1c, drinking status, smoking status, FPG and SBP.

Sensitivity-3 was adjusted for sex, BMI, ALT, AST, habit of exercise, GGT, HDL-C, TC, TG, MetS, HbA1c, drinking status, smoking status, FPG and SBP.

Sensitivity-3 was adjusted for sex, age, BMI, ALT, AST, habit of exercise, GGT, HDL-C, TC, TG, HbA1c, drinking status, smoking status, FPG and SBP.

### Nonlinearity relationship between BRI and MASLD

**Fig. 3:**
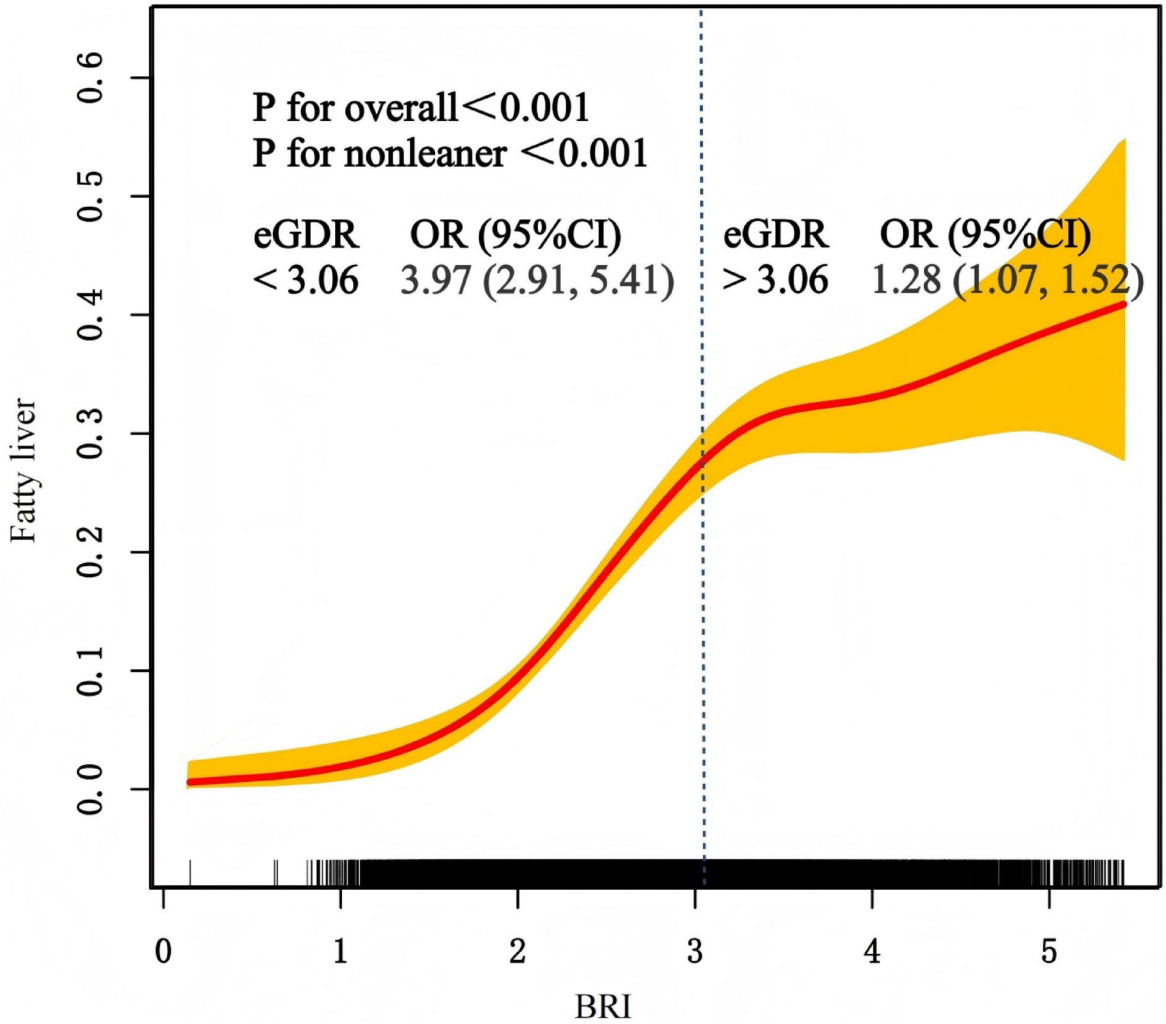
Restricted cubic spline analysis of BRI for estimating the risk of MASLD.

The analysis using a restricted cubic spline regression model revealed a significant nonlinear relationship between BRI and MASLD (Fig. 3). Threshold effect analysis determined that BRI = 3.06 serves as the critical inflection point in this relationship. When BRI values dropped below 3.06, the OR reached as high as 3.97 (95% CI 2.91–5.41). However, when BRI values exceeded this threshold, the OR decreased to 1.28 (95% CI 1.07–1.52).

## Discussion

This study examined the association between BRI and MASLD in non-diabetic Japanese adults using the NAGALA data, and identified three main findings. Firstly, an overall MASLD prevalence of 14.46% was observed, with a prevalence of 13.37% among the non-obese non-diabetic population (BMI < 30). Secondly, BRI showed a positive association with MASLD in non-diabetic Japanese adults, and the point of inflection was found to be 3.06. Thirdly, stratified analysis demonstrated that this association was stronger in individuals with a lower BMI.

The prevalence of MASLD varies significantly across different populations, with global rates reaching approximately 25% and specific rates of 23% in Germany^26^. The overall prevalence in Asia stands at 29.62%, with notable variation: Indonesia shows the highest prevalence at 51.04%, while Japan is the lowest at 22.28%^27^. In East Asia, the prevalence is around 29.7%, ranging from 25.95 to 33.76%, despite lower rates of overweight and obesity compared to Western countries^5^. A study of 831 faculty and staff at Gifu University undergoing annual occupational health examinations reported a MASLD prevalence of approximately 26% in the total group and 13% among those classified as non-obese (BMI < 25 kg/m^2^)^28^. This disparity indicates that Asian populations may have an elevated risk of MASLD, potentially influenced by genetic factors and dietary habits that that contribute to the onset and progression of the disease^29,30^. This study investigates the prevalence of MASLD in a non-diabetic population, identifying a notable prevalence rate of 14.46%. These findings highlight the urgent need to address MASLD in non-diabetic individuals and underscore the importance of further research targeting this specific population.

Recent studies on BRI and MASLD have highlighted both commonalities and differences across various countries. In a large community-based study involving 4872 participants in northern Iran, BRI showed a strong association with ultrasound-defined MASLD (OR 5.484 in men and OR 3.482 in women)^31^. In the United States, BRI was identified as a more effective predictor of MASLD than BMI^32^. Moreover, there is a nonlinear relationship between BRI and MASLD^33^. These findings highlight the importance of managing BRI levels to reduce MASLD incidence. Research from Taiwan and China also confirms a positive association between BRI and the severity and progression of MASLD^34^. This study further observed a nonlinear association within the non-diabetes Japanese population. Specifically, the risk of MASLD prevalence escalated rapidly with increasing BRI below 3.06, but the increase moderated when BRI exceeded 3.06. The nonlinear association between BRI and MASLD demonstrates a critical inflection point at BRI = 3.06, suggesting differential contributions of visceral fat to MASLD risk^35^. Below this threshold, the sharp increase in OR highlights the pivotal role of visceral fat in driving hepatic metabolic dysfunction. Beyond this inflection point, the plateau effect observed may reflect a state of metabolic saturation, where further visceral fat accumulation has a diminished impact on MASLD risk. Potential mechanisms underlying this phenomenon include genetic predispositions, systemic inflammation, or adaptive changes in hepatic lipid metabolism^36,37^. Unlike previous studies, our findings reveal that the association between BRI and MASLD varies significantly depending on body fat.

Body fat accumulation is a major pathophysiological factor in MASLD^38^. While obesity accelerates liver fibrosis in MASLD patients^39,40^. MASLD typically coincides with the disease, non-obese MASLD is also prevalent, with global rates around 12.1% and higher in countries like Austria, Mexico, and Sweden^41^. In East Asia, this prevalence ranges from approximately 7.2–27%^42^. Our cohort study revealed a 14.46% prevalence of MASLD, with 13.37% among non-obese individuals (BMI < 30). Additionally, there are notable racial differences in BMI and its correlation with MASLD; Asians have an elevated MASLD risk at lower BMI levels^43^. The Stratified analysis indicated that the relationship between BRI and MASLD was significantly stronger in participants with lower BMI compared to those with higher BMI. MI-based predictive indices, including the Fatty Liver Index and Hepatic Steatosis Index, are effective tools for detecting MASLD^44,45^. Genetic polymorphisms, such as in the PNPLA3 gene, influence MASLD progression across racial groups, indicating excessive visceral fat despite normal BMI^46^. Recent research highlights Golgi membrane protein 73 as a contributing factor, inhibiting lipid export from the liver and elevating disease risk, particularly in non-obese MASLD^47^. BMI is an established risk factor for MASLD; Interestingly, this research indicated that individuals with a BMI below 24 exhibited a stronger association between BRI and MASLD. This finding suggests that BRI may be particularly informative in East Asian populations, who are more susceptible to genetic and weight-related factors^33^.

Additionally, MASLD differs from NAFLD by focusing on metabolic dysfunction as the central pathological driver^48^. MetS contributes to varying degrees of impact on cardiovascular outcomes in individuals with MASLD^49^. Fortunately, this research found that MetS did not affect the association between BRI and MASLD. Moreover, the results of sensitivity analyses further support our conclusions.

### Limitations

This study represents the first comprehensive cohort investigation of the relationship between BRI and MASLD in a non-diabetic Japanese population. Nevertheless, it is crucial to acknowledge the various constraints inherent in the design of our study. Firstly, although we thoroughly gathered potential confounding variables, we overlooked important factors like dietary patterns, stress, and genetic makeup that may influence the development of MASLD. Secondly, the diagnosis of MASLD was based on ultrasonography, which cannot determine disease progression and may underestimate its prevalence. Thirdly, this study excluded individuals with diabetes and included a limited percentage of elderly participants, which may affect the generalizability of the findings to broader populations. Forthly, the small number of individuals with obesity in the sample limits its representativeness, making it challenging to determine whether the index provides added value over established indicators of body fat or offers clinical relevance and additional predictive utility. Finally, this study did not include FLI and HSI indices, and future research with larger cohorts is needed to validate the comparative efficacy of BRI against these indices. Future research should incorporate more objective measurement methods to enhance the reliability and validity of conclusions.

## Conclusion

This study established a significant nonlinear relationship between BRI and MASLD in a non-diabetic Japanese adult population, with a particularly strong association observed in individuals with lower BMI. Considering the limitations inherent to the study population, future research should aim to increase the sample size and incorporate multicenter cohorts representing diverse ethnicities and geographic regions to further validate the utility of BRI in predicting MASLD across a wider demographic.

## Electronic supplementary material

Below is the link to the electronic supplementary material.

Supplementary Material 1

## Data Availability

The raw data can be downloaded from the ‘DATADRYAD’ database (www.Datadryad.org). https://datadryad.org/stash/dataset/doi:10.5061%2Fdryad.8q0p192.

## References

[1] Eslam, M., Sanyal, A. J. & George, J. MAFLD: A consensus-driven proposed nomenclature for metabolic associated fatty liver disease. *Gastroenterology* **158**, 1999–2014. 10.1053/j.gastro.2019.11.312 (2020). 32044314 10.1053/j.gastro.2019.11.312

[2] Targher, G., Byrne, C. D. & Tilg, H. MASLD: A systemic metabolic disorder with cardiovascular and malignant complications. *Gut* **73**, 691–702. 10.1136/gutjnl-2023-330595 (2024). 38228377 10.1136/gutjnl-2023-330595

[3] Yang, A., Zhu, X., Zhang, L. & Ding, Y. Transitioning from NAFLD to MAFLD and MASLD: Consistent prevalence and risk factors in a Chinese cohort. *J. Hepatol.* **80**, e154–e155. 10.1016/j.jhep.2023.09.033 (2024). 37827472 10.1016/j.jhep.2023.09.033

[4] Wong, V.W.-S., Ekstedt, M., Wong, G.L.-H. & Hagström, H. Changing epidemiology, global trends and implications for outcomes of NAFLD. *J. Hepatol.* **79**, 842–852. 10.1016/j.jhep.2023.04.036 (2023). 37169151 10.1016/j.jhep.2023.04.036

[5] Younossi, Z. M. et al. The global epidemiology of nonalcoholic fatty liver disease (NAFLD) and nonalcoholic steatohepatitis (NASH): A systematic review. *Hepatology* **77**, 1335–1347. 10.1097/HEP.0000000000000004 (2023). 36626630 10.1097/HEP.0000000000000004 PMC10026948

[6] Paik, J. M. et al. Global burden of NAFLD and chronic liver disease among adolescents and young adults. *Hepatology* **75**, 1204–1217. 10.1002/hep.32228 (2022). 34741554 10.1002/hep.32228

[7] Lonardo, A. et al. Metabolic mechanisms for and treatment of NAFLD or NASH occurring after liver transplantation. *Nat. Rev. Endocrinol.* **18**, 638–650. 10.1038/s41574-022-00711-5 (2022). 35840803 10.1038/s41574-022-00711-5

[8] Younossi, Z. et al. Global burden of NAFLD and NASH: Trends, predictions, risk factors and prevention. *Nat. Rev. Gastroenterol. Hepatol.* **15**, 11–20. 10.1038/nrgastro.2017.109 (2018). 28930295 10.1038/nrgastro.2017.109

[9] Yahoo, N., Dudek, M., Knolle, P. & Heikenwälder, M. Role of immune responses in the development of NAFLD-associated liver cancer and prospects for therapeutic modulation. *J. Hepatol.* **79**, 538–551. 10.1016/j.jhep.2023.02.033 (2023). 36893854 10.1016/j.jhep.2023.02.033

[10] Caussy, C., Aubin, A. & Loomba, R. The relationship between type 2 diabetes, NAFLD, and cardiovascular risk. *Curr. Diabetes Rep.* **21**, 15. 10.1007/s11892-021-01383-7 (2021). 10.1007/s11892-021-01383-7 PMC8805985 33742318

[11] Kim, D., Chung, G. E., Kwak, M.-S., Kim, Y. J. & Yoon, J.-H. Effect of longitudinal changes of body fat on the incidence and regression of nonalcoholic fatty liver disease. *Dig. Liver Dis. Off. J. Ital. Soc. Gastroenterol. Ital. Assoc. Study Liver* **50**, 389–395. 10.1016/j.dld.2017.12.014 (2018). 10.1016/j.dld.2017.12.014 29373238

[12] Ji, L., Cai, X., Bai, Y. & Li, T. Application of a novel prediction model for predicting 2-year risk of non-alcoholic fatty liver disease in the non-obese population with normal blood lipid levels: A Large prospective cohort study from China. *Int. J. Gen. Med.* **14**, 2909–2922. 10.2147/IJGM.S319759 (2021). 34234521 10.2147/IJGM.S319759 PMC8254414

[13] Parsa, A. A., Azama, K. A., Vawer, M., Ona, M. A. & Seto, T. B. Prevalence study of MASLD in adolescent and young adult Pacific Islanders and Asians living in Hawai’i. *J. Endocr. Soc.* **8**, bvad165. 10.1210/jendso/bvad165 (2024). 38249431 10.1210/jendso/bvad165 PMC10797323

[14] Thomas, D. M. et al. Relationships between body roundness with body fat and visceral adipose tissue emerging from a new geometrical model. *Obesity (Silver, Spring, Md.)* **21**, 2264–2271. 10.1002/oby.20408 (2013). 23519954 10.1002/oby.20408 PMC3692604

[15] Rico-Martín, S. et al. Effectiveness of body roundness index in predicting metabolic syndrome: A systematic review and meta-analysis. *Obes. Rev.* **21**, e13023. 10.1111/obr.13023 (2020). 32267621 10.1111/obr.13023

[16] Li, Y. et al. Body roundness index and waist-hip ratio result in better cardiovascular disease risk stratification: Results from a large Chinese cross-sectional study. *Front. Nutr.* **9**, 801582. 10.3389/fnut.2022.801582 (2022). 35360688 10.3389/fnut.2022.801582 PMC8960742

[17] Li, Z. et al. Non-linear relationship between the body roundness index and metabolic syndrome: Data from National Health and Nutrition Examination Survey (NHANES) 1999–2018. *Br. J. Nutr.* **131**, 1852–1859. 10.1017/S0007114524000357 (2024). 38356387 10.1017/S0007114524000357

[18] Zhang, X. et al. Body roundness index and all-cause mortality among US adults. *JAMA Netw. Open* **7**, e2415051. 10.1001/jamanetworkopen.2024.15051 (2024). 38837158 10.1001/jamanetworkopen.2024.15051 PMC11154161

[19] Jiang, N., Zhang, S., Chu, J., Yang, N. & Lu, M. Association between body roundness index and non-alcoholic fatty liver disease detected by Fibroscan in America. *J. Clin. Lab. Anal.* **37**, e24973. 10.1002/jcla.24973 (2023). 37850486 10.1002/jcla.24973 PMC10681427

[20] Okamura, T. et al. Ectopic fat obesity presents the greatest risk for incident type 2 diabetes: A population-based longitudinal study. *Int. J. Obes.* **2005**(43), 139–148. 10.1038/s41366-018-0076-3 (2019). 10.1038/s41366-018-0076-3 29717276

[21] Hashimoto, Y. et al. Modest alcohol consumption reduces the incidence of fatty liver in men: A population-based large-scale cohort study. *J. Gastroenterol. Hepatol.* **30**, 546–552. 10.1111/jgh.12786 (2015). 25238605 10.1111/jgh.12786

[22] Hamaguchi, M. et al. The severity of ultrasonographic findings in nonalcoholic fatty liver disease reflects the metabolic syndrome and visceral fat accumulation. *Am. J. Gastroenterol.* **102**, 2708–2715 (2007). 17894848 10.1111/j.1572-0241.2007.01526.x

[23] Li, W. et al. Association between metabolic syndrome and mortality: Prospective cohort study. *JMIR Public Health Surveill.* **9**, e44073. 10.2196/44073 (2023). 37669100 10.2196/44073 PMC10509744

[24] Eslam, M. et al. A new definition for metabolic dysfunction-associated fatty liver disease: An international expert consensus statement. *J. Hepatol.* **73**, 202–209. 10.1016/j.jhep.2020.03.039 (2020). 32278004 10.1016/j.jhep.2020.03.039

[25] Shen, D. et al. Associating plasma aldosterone concentration with the prevalence of MAFLD in hypertensive patients: Insights from a large-scale cross-sectional study. *Front. Endocrinol. (Lausanne)* **15**, 1451383. 10.3389/fendo.2024.1451383 (2024). 39363897 10.3389/fendo.2024.1451383 PMC11446807

[26] Updated S2k clinical practice guideline on non-alcoholic fatty liver disease (NAFLD) issued by the German Society of Gastroenterology, Digestive and Metabolic Diseases (DGVS)—April 2022—AWMF Registration No.: 021–025. *Zeitschrift Fur Gastroenterologie* **60**, e733-e801. 10.1055/a-1880-2388 (2022). 10.1055/a-1880-2388 36100201

[27] Li, J. et al. Prevalence, incidence, and outcome of non-alcoholic fatty liver disease in Asia, 1999–2019: A systematic review and meta-analysis. *Lancet Gastroenterol. Hepatol.* **4**, 389–398. 10.1016/S2468-1253(19)30039-1 (2019). 30902670 10.1016/S2468-1253(19)30039-1

[28] Miwa, T. et al. Prevalence of steatotic liver disease based on a new nomenclature in the Japanese population: A health checkup-based cross-sectional study. *J. Clin. Med.* **13**, 1158. 10.3390/jcm13041158 (2024). 38398471 10.3390/jcm13041158 PMC10888602

[29] Miwa, T. et al. Usefulness of a questionnaire for assessing the relationship between eating behavior and steatotic liver disease among Japanese male young adults. *Sci. Rep.* **14**, 2194. 10.1038/s41598-024-52797-8 (2024). 38273030 10.1038/s41598-024-52797-8 PMC10810865

[30] Li, L. et al. Obesity is an independent risk factor for non-alcoholic fatty liver disease: Evidence from a meta-analysis of 21 cohort studies. *Obes. Rev.* **17**, 510–519. 10.1111/obr.12407 (2016). 27020692 10.1111/obr.12407

[31] Motamed, N. et al. Body roundness index and waist-to-height ratio are strongly associated with non-alcoholic fatty liver disease: A population-based study. *Hepat. Mon.* **16**, e39575 (2016). 27822266 10.5812/hepatmon.39575 PMC5091031

[32] Tian, X., Ding, N., Su, Y. & Qin, J. Comparison of obesity-related indicators for nonalcoholic fatty liver disease diagnosed by transient elastography. *Turk. J. Gastroenterol. Off. J. Turk. Soc. Gastroenterol.* **34**, 1078–1087. 10.5152/tjg.2023.23101 (2023). 10.5152/tjg.2023.23101 PMC10645279 37737216

[33] Zhao, E., Wen, X., Qiu, W. & Zhang, C. Association between body roundness index and risk of ultrasound-defined non-alcoholic fatty liver disease. *Heliyon* **10**, e23429. 10.1016/j.heliyon.2023.e23429 (2024). 38170062 10.1016/j.heliyon.2023.e23429 PMC10758814

[34] Lin, I. T., Lee, M.-Y., Wang, C.-W., Wu, D.-W. & Chen, S.-C. Gender differences in the relationships among metabolic syndrome and various obesity-related indices with nonalcoholic fatty liver disease in a Taiwanese population. *Int. J. Environ. Res. Public Health* **18**, 857. 10.3390/ijerph18030857 (2021). 33498329 10.3390/ijerph18030857 PMC7908550

[35] Sun, Y. et al. Autoimmune mechanisms and inflammation in obesity-associated type 2 diabetes, atherosclerosis, and non-alcoholic fatty liver disease. *Funct. Integr. Genom.* **25**, 84. 10.1007/s10142-025-01587-0 (2025). 10.1007/s10142-025-01587-0 40205260

[36] Shen, D. et al. Inflammatory indices and MAFLD prevalence in hypertensive patients: A large-scale cross-sectional analysis from China. *J. Inflamm. Res.* **18**, 1623–1638. 10.2147/JIR.S503648 (2025). 39925928 10.2147/JIR.S503648 PMC11806676

[37] Hu, J. et al. Relationship between plasma aldosterone concentrations and non-alcoholic fatty liver disease diagnosis in patients with hypertension: A retrospective cohort study. *Diabetes Metab. Syndr. Obes.* **16**, 1625–1636. 10.2147/DMSO.S408722 (2023). 37304667 10.2147/DMSO.S408722 PMC10257476

[38] Miwa, T. et al. Impact of body fat accumulation on metabolic dysfunction-associated fatty liver disease and nonalcoholic fatty liver disease in Japanese male young adults. *Hepatol. Res.* **53**, 691–700. 10.1111/hepr.13906 (2023). 37143429 10.1111/hepr.13906

[39] Estes, C. et al. Modeling NAFLD disease burden in China, France, Germany, Italy, Japan, Spain, United Kingdom, and United States for the period 2016–2030. *J. Hepatol.* **69**, 896–904. 10.1016/j.jhep.2018.05.036 (2018). 29886156 10.1016/j.jhep.2018.05.036

[40] Lin, C.-L. et al. The impact of body mass index on clinicopathological features of nonalcoholic fatty liver disease in Taiwan. *J. Gastroenterol. Hepatol.* **37**, 1901–1910. 10.1111/jgh.15936 (2022). 35790343 10.1111/jgh.15936

[41] Ye, Q. et al. Global prevalence, incidence, and outcomes of non-obese or lean non-alcoholic fatty liver disease: A systematic review and meta-analysis. *Lancet Gastroenterol. Hepatol.* **5**, 739–752. 10.1016/S2468-1253(20)30077-7 (2020). 32413340 10.1016/S2468-1253(20)30077-7

[42] Kumar, R. & Mohan, S. Non-alcoholic fatty liver disease in lean subjects: Characteristics and implications. *J. Clin. Transl. Hepatol.* **5**, 216–223 (2017). 28936403 10.14218/JCTH.2016.00068 PMC5606968

[43] Zhou, J. et al. Epidemiological features of NAFLD from 1999 to 2018 in China. *Hepatology* **71**, 1851–1864. 10.1002/hep.31150 (2020). 32012320 10.1002/hep.31150

[44] Kaneva, A. M. & Bojko, E. R. Fatty liver index (FLI): More than a marker of hepatic steatosis. *J. Physiol. Biochem.* **80**, 11–26. 10.1007/s13105-023-00991-z (2024). 37875710 10.1007/s13105-023-00991-z

[45] Xie, R. & Zhang, Y. Is assessing the degree of hepatic steatosis and fibrosis based on index calculations the best choice for epidemiological studies?. *Environ. Pollut.* **317**, 120783. 10.1016/j.envpol.2022.120783 (2023). 36460186 10.1016/j.envpol.2022.120783

[46] Cherubini, A., Casirati, E., Tomasi, M. & Valenti, L. PNPLA3 as a therapeutic target for fatty liver disease: The evidence to date. *Expert Opin. Ther. Targets* **25**, 1033–1043. 10.1080/14728222.2021.2018418 (2021). 34904923 10.1080/14728222.2021.2018418

[47] Li, Y. et al. Use of GP73 in the diagnosis of non-alcoholic steatohepatitis and the staging of hepatic fibrosis. *J. Int. Med. Res.* **49**, 3000605211055378. 10.1177/03000605211055378 (2021). 34772312 10.1177/03000605211055378 PMC8593324

[48] Hutchison, A. L., Tavaglione, F., Romeo, S. & Charlton, M. Endocrine aspects of metabolic dysfunction-associated steatotic liver disease (MASLD): Beyond insulin resistance. *J. Hepatol.* **79**, 1524–1541. 10.1016/j.jhep.2023.08.030 (2023). 37730124 10.1016/j.jhep.2023.08.030

[49] Elsaid, M. I. et al. The impact of metabolic syndrome severity on racial and ethnic disparities in metabolic dysfunction-associated steatotic liver disease. *PLoS ONE* **19**, e0299836. 10.1371/journal.pone.0299836 (2024). 38489287 10.1371/journal.pone.0299836 PMC10942082

