# Supplementary material for: Nonlinear association between body roundness index and metabolic dysfunction associated steatotic liver disease in nondiabetic Japanese adults
Source: Sci Rep. 2025 May 2;15:15442. doi: 10.1038/s41598-025-99540-5 (PMC12048529; doi:10.1038/s41598-025-99540-5)
Supplement: Supplementary file 1 — Supplementary Material 1 [file 41598_2025_99540_MOESM1_ESM.doc]

**Table S1: Collinearity screening between BRI and other variables.**

|  | Variance inflation factor | |
| --- | --- | --- |
|  | Step 1 | Step 2 |
| BRI | 4.1 | 4.1 |
| Sex (M/F) | 2.1 | 2.1 |
| Age, years | 1.3 | 1.3 |
| BMI (kg/m^2^) | 4.4 | 4.4 |
| ALT (IU/L) | 3.2 | 3.2 |
| AST (IU/L) | 3.8 | 3.8 |
| Habit of exercise | 1 | 1 |
| GGT (IU/L) | 1.5 | 1.5 |
| HDL-C (mmol/L) | 1.8 | 1.8 |
| TC (mmol/L) | 1.4 | 1.4 |
| TG (mmol/L) | 1.9 | 1.9 |
| HbA1c, % | 1.2 | 1.2 |
| Drinking status, n (%) | 1.3 | 1.3 |
| Smoking status, n (%) | 1.4 | 1.4 |
| FPG (mmol/L) | 1.5 | 1.5 |
| SBP (mmHg) | 5.5 | 1.4 |
| DBP (mmHg) | 5.7 | NA |
| METS | 1.3 | 1.3 |

Note-1: Variance inflation factor = 1/(1-R^2^). Abbreviations as in Table 1.

Note-2: The variables with variance inflation factor >5 will be regarded as collinear variables and cannot be included in the multiple regression model.

Variance inflation factor

**Table S2: Univariate regression analysis of the associations between MASLD** and baseline variables.

|  | **Statistics** | **OR (95%CI)** | ***P*-value** |
| --- | --- | --- | --- |
| Male, n (%) | 8353 (54.60%) | 5.10 (4.54, 5.74) | <0.0001 |
| Age (years) | 43.70 ± 8.89 | 1.02 (1.01, 1.02) | <0.0001 |
| BMI (kg/m^2^) | 22.02 ± 2.96 | 1.77 (1.73, 1.81) | <0.0001 |
| WC (cm) | 76.21 ± 8.74 | 1.23 (1.22, 1.24) | <0.0001 |
| SBP (mmHg) | 114.30 ± 14.85 | 1.06 (1.05, 1.06) | <0.0001 |
| DBP (mmHg) | 71.46 ± 10.45 | 1.08 (1.08, 1.09) | <0.0001 |
| BRI | 2.71 ± 0.83 | 5.66 (5.27, 6.09) | <0.0001 |
| MetS, n (%) | 571 (3.73%) | 12.45 (10.42, 14.87) | <0.001 |
| ALT (IU/L) | 19.83 ± 14.05 | 1.09 (1.09, 1.10) | <0.0001 |
| AST (IU/L) | 18.33 ± 8.50 | 1.09 (1.08, 1.09) | <0.0001 |
| Habit of exercise, n (%) | 2693 (17.60%) | 0.76 (0.67, 0.86) | <0.0001 |
| GGT (IU/L) | 20.21 ± 18.00 | 1.03 (1.03, 1.03) | <0.0001 |
| HDL-C (mmol/L) | 1.46 ± 0.40 | 0.04 (0.03, 0.05) | <0.0001 |
| TC (mmol/L) | 5.12 ± 0.86 | 1.68 (1.59, 1.76) | <0.0001 |
| TG (mmol/L) | 0.91 ± 0.65 | 4.49 (4.16, 4.84) | <0.0001 |
| FPG (mmol/L) | 5.16 ± 0.41 | 9.05 (7.96, 10.28) | <0.0001 |
| HbA1c (%) | 5.17 ± 0.32 | 4.91 (4.25, 5.68) | <0.0001 |
| Drinking status, n (%) |  |  |  |
| Non | 11671 (76.29%) | Ref |  |
| Light | 1740 (11.37%) | 0.92 (0.79, 1.07) | 0.2729 |
| moderate | 1350 (8.82%) | 1.13 (0.97, 1.32) | 0.1146 |
| heavy | 538 (3.52%) | 1.44 (1.16, 1.80) | 0.0011 |
| Smoking status, n (%) |  |  |  |
| Never | 8924 (58.33%) | Ref |  |
| Past | 2935 (19.18%) | 2.15 (1.92, 2.40) | <0.0001 |
| Current | 3440 (22.49%) | 1.95 (1.75, 2.18) | <0.0001 |

Abbreviations: ALT, alanine aminotransferase; AST, aspartate aminotransferase; BMI, body mass index; TC, total cholesterol; TG, triglycerides; HDL-C, high-density lipoprotein cholesterol; HbA1c, glycated hemoglobin; FPG, fasting plasma glucose; SBP, systolic blood pressure; DBP, diastolic blood pressure; MetS, Metabolic Syndrome; MASLD, metabolic dysfunction associated steatotic liver disease.

**Table S3. Multivariable regression analyses for the association between BRI and MASLD with extreme BRI values.**

|  |  | |  | **OR (95%CI)** | |  |  |
| --- | --- | --- | --- | --- | --- | --- | --- |
|  | **Model 1** |  |  | **Model 2** |  |  | **Model 3** |
| BRI | 1.99 (1.77, 2.25) |  |  | 1.70 (1.50, 1.93) |  |  | 1.58 (1.39, 1.79) |
| Q1 | Ref |  |  | Ref |  |  | Ref |
| Q2 | 3.83 (2.60, 5.65) |  |  | 3.52 (2.37, 5.21) |  |  | 3.05 (2.05, 4.53) |
| Q3 | 7.40 (5.03, 10.86) |  |  | 6.10 (4.13, 9.02) |  |  | 4.78 (3.23, 7.09) |
| Q4 | 11.05 (7.36, 16.59) |  |  | 8.17 (5.40, 12.37) |  |  | 6.13 (4.03, 9.31) |
| *P*-trend | <0.001 |  |  | <0.001 |  |  | <0.001 |

This analysis includes 15,453 participants, incorporating extreme BRI values (defined as BRI < mean - 3*SD or BRI > mean + 3*SD).

Model 1: sex, age, BMI, drinking status, smoking status, habit of exercise and SBP were adjusted.

Model 2: sex, age, BMI, ALT, AST, GGT, habit of exercise, HbA1c, drinking status, smoking status, FPG and SBP were adjusted.

Adjust 3: sex, age, BMI, ALT, AST, habit of exercise, GGT, HDL-C, TC, TG, MetS, HbA1c, drinking status, smoking status, FPG and SBP were adjusted.
